# The Quality Management Improvement Approach: Successes and Lessons Learned From a Workforce Development Intervention in Rwanda’s Health Supply Chain

**DOI:** 10.9745/GHSP-D-22-00295

**Published:** 2023-02-28

**Authors:** Rogers Kigenza, Eliezer Nsengiyumva, Vincent Sabagirirwa

**Affiliations:** a U.S. Agency for International Development Global Health Supply Chain Program-Procurement and Supply Management, Kigali, Rwanda.; b Clinical and Public Health Governance Directorate, Ministry of Health of Rwanda, Kigali, Rwanda.

## Abstract

**Background:**

Rwanda’s supply chain is decentralized, with regional warehouse pharmacies and service delivery points (SDPs) ensuring a reliable health commodities supply. However, in 2016, the Rwanda Ministry of Health (MOH) identified challenges with the supply chain workforce contributing to a high stock-out rate, inventory stock inaccuracies, and underutilization of the electronic logistics management information system (e-LMIS). We aimed to document successes and lessons learned from implementing a workforce development intervention to address these challenges.

**Program Description:**

Rwanda’s MOH and the U.S. Agency for International Development Global Health Supply Chain Program-Procurement and Supply Management project implemented a workforce development intervention, the Quality Management Improvement Approach (QMIA), to improve and sustain Rwanda’s service delivery and medicines availability. The QMIA monitors the performance of supply chain professionals while continuously building their capacity to run a smooth supply chain operation. The QMIA includes supervisory visits to measure key performance indicators and discussion sessions for experience-sharing and skill-building. The central level conducts both components, supporting regional warehouses that in turn assist SDPs through supervisory visits and discussion sessions.

**Results:**

Since 2017, the QMIA has been conducted twice a year in 596 SDPs and 30 regional warehouses and has trained 1,296 supply chain staff members. The intervention has contributed to major improvements in several key supply chain outcomes (2017 versus 2019): increased utilization of the e-LMIS (55% to 96%), higher inventory data accuracy (25% to 85%), and reduced stock-outs (10% to 1%).

**Conclusions:**

The QMIA has proven to be a successful training and capacity-building platform for supply chain management in Rwanda, enhancing system utilization and availability of accurate data to guide decision-making. Notably, the approach fosters a positive behavioral change to build local solutions and improves communication.

## Abstract (precis)

A workforce development intervention called the Quality Management Improvement Approach has proven to be a successful training and capacity-building platform for supply chain management, improving end-to-end data visibility and communication.

## Teaser

**Key Findings:**

- High staff turnover, limited training, and lack of capacity for supply chain activities contributed to challenges in the supply chain management system, including stock-outs and stock inaccuracies.
- The Quality Management Improvement Approach contributed to significant improvements in staff recording of commodity consumption, using the electronic logistics management information system, and keeping accurate inventory data, as well as reduced stock-outs for all types of commodities.
- The approach addressed performance gaps created by staff turnover, fostered an improvement in communication between supply chain workers at different levels, and facilitated developing local solutions to resolve issues and challenges in supply chain operation and management.

## BACKGROUND

Over the last few years, the Government of Rwanda has invested significantly in improving the country’s health care system, including the accessibility and availability of essential health commodities to its citizens.^1^ As a result, quality control, storage capacity, and rational use of health commodities have greatly improved in the country.^2^ However, the Rwanda Health Sector Strategic Plan 2018–2024 (HSSP IV)^2^ still recognizes the limited capacity of supply chain management at different levels as a crucial challenge. Thus, strengthening the capacity in supply chain management systems, including storage conditions, human resources, financing, supply plans, and supply regulations, together with upgrading information and technology tools, constitute a national strategic direction.

Rwanda has a well-structured supply chain management system that is decentralized from the Ministry of Health (MOH). The MOH coordinates all the supply chain operations through the Logistics Management Office. Rwanda Food and Drug Authority regulates all the processes for pharmaceutical management to ensure quality and safety, using the Pharmaceutical Regulatory Information Management System to operate. Rwanda Biomedical Center promotes high-quality, affordable, and sustainable health care services to the population through evidence-based interventions and practices.

**FIGURE 1:**
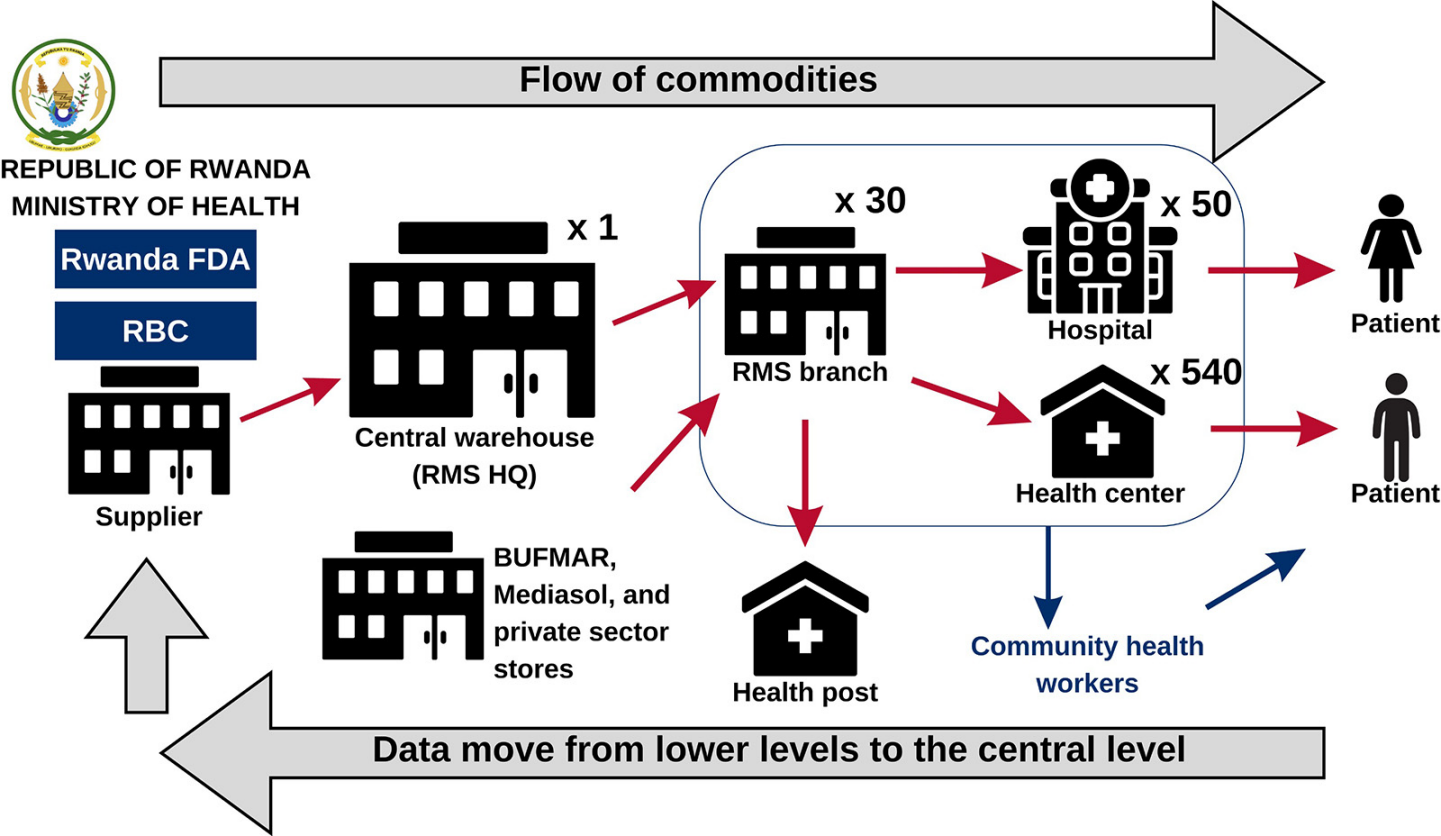
Commodity and Data Flow in Rwanda’s Health Supply Chain Abbreviations: BUFMAR, Bureau des Formations Médicales Agréées du Rwanda; FDA, Food and Drug Authority; HQ, headquarters; Mediasol, Medical & Allied Service Solutions; RBC, Rwanda Biomedical Center; RMS, Rwanda Medical Supply.

Currently, the country uses a “pull system” for all product categories where data moves in a reverse direction of the commodities (Figure 1).^3^ The supply chain system is managed by the Rwanda Medical Supply (RMS), a large-scale corporation created and owned by the Government of Rwanda to ensure the availability of medicines and health commodities and overcome past problems of low availability at the public central medical store (called the Medical Production and Procurement Division).^4^

The RMS, Bureau des Formations Médicales Agréées du Rwanda, Medical & Allied Service Solutions, and the private-sector stores operate as central medical stores to ensure the procurement and distribution of health commodities using the electronic logistics management information system (e-LMIS) and warehouse management system. Supply chain master data include facility master data and national product master data.

The RMS headquarters in Kigali is where senior management and a central warehouse are located. The central warehouse supplies the RMS branches, which are district-level warehouses that supply service delivery points (SDPs), including hospitals and health centers. The 30 RMS branches located in 30 districts (1 RMS branch per district) play a key role in storage and inventory management, transportation of commodities to health facilities, resupply, and support to and supervision of SDPs within their respective districts. Together with health posts and community health workers, the system ensures that medicines and supplies reach patients in a timely manner and in the right quantity, condition, and cost. RMS branches use the e-LMIS launched in 2014.^5^ Therefore, any supply chain performance improvement at lower levels will depend on how well the RMS branches perform.

In 2016, the MOH conducted supply chain monitoring, training, and planning sessions in collaboration with the U.S. Agency for International Development Global Health Supply Chain Program-Procurement and Supply Management (GHSC-PSM) project. These sessions identified several challenges related to the supply chain workforce, such as high staff turnover (in some cases more than 30% due to excessive workloads, lack of training, or staff receiving better offers elsewhere), minimal time dedicated to supply chain activities at SDPs (e.g., nurses already having other work assignments), and lack of supply chain professionals at the SDP level, among others.^6^ These challenges resulted in a high stock-out rate (10%), underutilization of the e-LMIS (55%), and inventory stock inaccuracies (25%), as reported by SDPs and district pharmacies.^6^ The MOH and GHSC-PSM concluded that the key to successfully improving supply chain performance was to focus not only on areas that were underperforming but also on areas that were not aligned with the overall supply chain strategy.^6^

Challenges with high staff turnover and lack of capacity for supply chain activities led to the need for a strategy to improve supply chain professionals’ performance.

GHSC-PSM offered procurement and technical assistance in Rwanda to help ensure uninterrupted availability of high-quality health commodities, focusing on those to prevent and treat HIV/AIDS; malaria; family planning; and maternal, neonatal, child, and community health.^7^ In particular, since 2017, GHSC-PSM has supported the MOH in strengthening the last-mile capacity-building system using the Quality Management Improvement Approach (QMIA). This approach is a workforce development intervention the MOH uses to monitor the performance of supply chain professionals while continuously building their capacity to run a smooth supply chain operation.

We describe the successes and lessons learned from implementing the QMIA and offer recommendations for different levels of the supply chain management system.

## IMPLEMENTING QMIA

Quality improvement practices have been helpful in improving supply chain operations and access to medicines, as previously shown by an intervention establishing resupply procedures and quality improvement teams in Malawi and Rwanda.^8^ In Uganda, a strategy based on performance assessment and recognition effectively built supply chain management capacity in lower-level health care facilities and improved supply chain management.^9^

Thus, to solve the identified challenges related to the supply chain workforce in Rwanda, the MOH and GHSC-PSM aimed to establish a workforce development intervention called the QMIA. They defined the QMIA concept for the public health supply chain in Rwanda as “a participative, continuous, patient-centered improvement approach that stresses total staff commitment to customer/patient satisfaction in terms of medicines availability and quality by creating and implementing process improvement.”^10^ The approach is considered patient centered because it aims to ensure that patients get efficient service delivery in terms of time and quality. For example, patients provide feedback on time spent at hospitals and SDPs (before and after QMIA, although not a specific indicator of this activity).

### QMIA Guiding Principles and Objectives

**FIGURE 2:**
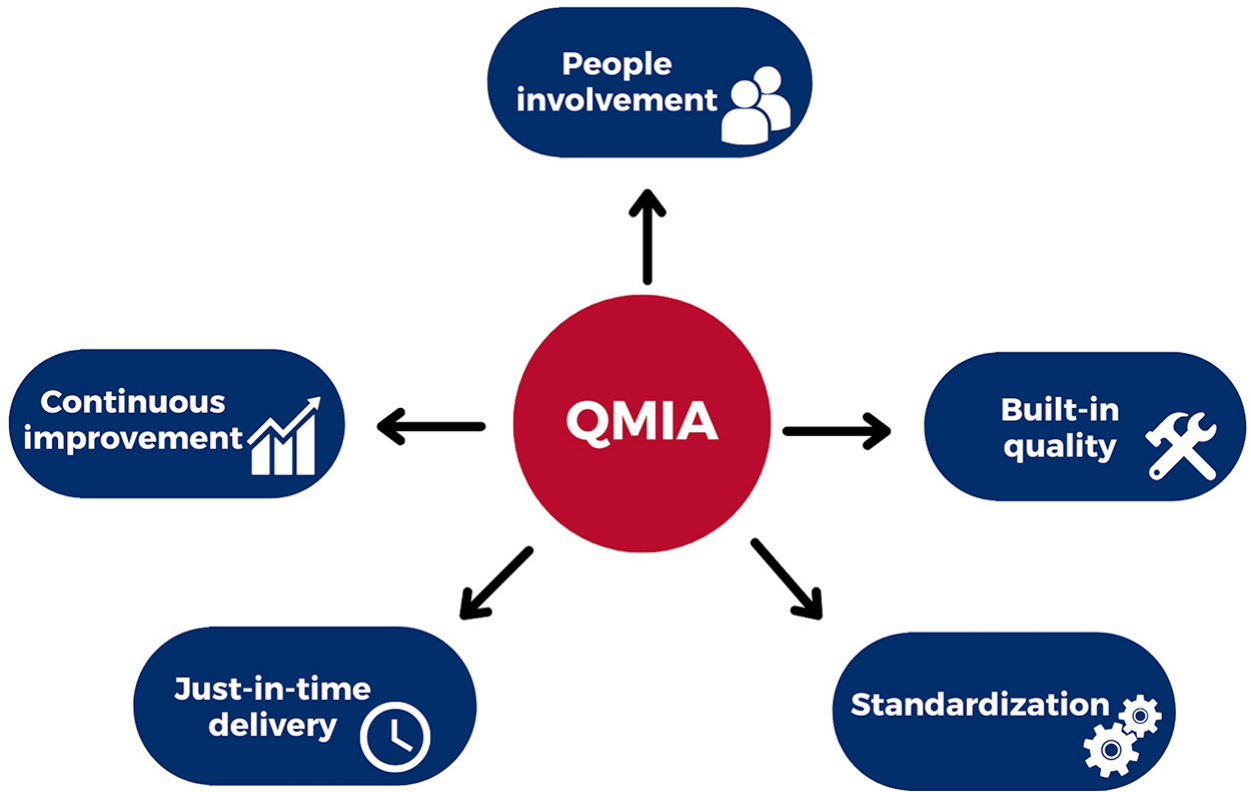
Five Guiding Principles of the Quality Management Improvement Approach Abbreviation: QMIA, Quality Management Improvement Approach.

The QMIA is based on a set of guiding principles^10^ (Figure 2): people involvement, based on the principles of teamwork, empowerment, and accountability; built-in quality (“do it right the first time, eliminate rework”); standardization (documenting best practices and establishing standard operating procedures); just-in-time delivery (ensuring the “7 rights of supply chain management” using an established inventory flow and pull system for reordering of health commodities); and continuous improvement (“every aspect should be challenged to get better”).^10^

The QMIA’s primary objective was to ensure continuous performance improvement and prevention of supply chain issues that may result in stock-outs, expiries, and overstocks at both RMS branches and SDPs (Box 1).

BOX 1 **Specific Objectives of the Quality Management Improvement Approach for Health Supply Chain Management in Rwanda**

- Ensure an uninterrupted supply of health commodities at both Rwanda Medical Supply branches and service delivery points
- Encourage a proper working environment and empower the staff to motivate them to improve services to patients/customers and foster their will to innovate and act in an atmosphere of trust and respect
- Share experiential knowledge on problem solving and problem prevention with other facilities
- Use measurements to support decisions (using measurements, the staff can spot trends, make corrections before problems arise and, as part of problem solving, investigate why problems happened and what can be done to prevent them from happening again)
- Provide capacity-building and supervision of staff

### Techniques and Tools Used for Process Improvement

To achieve its objectives, the QMIA is based on the Lean Six Sigma and the hybrid Lean Six Sigma methodologies, tailored for best-in-class supply executions, continuous improvement, and supply chain efficiency at all levels of the supply chain in Rwanda. The utility of applying these methods has been demonstrated in the context of health care logistics, adding value to the availability of health commodities.^11^^–^^14^ By relying on a collaborative team effort, MOH and GHSC-PSM used the QMIA to apply these methodologies to improve supply chain performance by systematically removing waste and reducing variation.

### Roles of Partners and Estimated Costs of the Approach

GHSC-PSM provided financial support to implement the activity. On average, the costs of implementing this approach were approximately US$300,000 per year during the first 2 years. Because the need for training workshops and continuous supervision has diminished in subsequent years, the costs have decreased to an estimated US$100,000–US$150,000 per year. The estimated cost for continuous implementation of the program is expected to be also around US$100,000–US$150,000 per year.

In addition, GHSC-PSM worked in conjunction with MOH staff to conduct field supervisory visits and data collection to evaluate improvements in service delivery at SDPs and RMS branches; analyzed and reported data for dissemination to the MOH; trained staff on QMIA at SDPs; mentored and advised staff at SDPs and RMS branches on areas that deserve improvement (using the information collected from previous visits); and participated in QMIA sessions together with MOH staff. With the overall oversight of the MOH, GHSC-PSM continues to provide technical assistance to build the necessary capacity and support the RMS branches to effectively supervise lower-level health facilities but also mentor them to measure and improve their performance. The participating staff are paid by their respective institutions and do not receive any economic incentive to participate.

### QMIA Intervention Areas and Components

The QMIA includes continuous capacity-building through mentorship, supervision, and performance improvement and measurement. The intervention also provides on-the-job mentorship to health facility staff regarding the planning and implementing of improvements in supply chain components, such as warehousing and inventory control, resupply, storage, waste management, data management and recordkeeping using the e-LMIS, pest control, and transportation, among others. The QMIA sessions have covered various key functional and cross-cutting supply chain areas (Box 2).

The QMIA includes continuous capacity-building through mentorship, supervision, and performance improvement and measurement.

BOX 2 **Intervention Areas of the Quality Management Improvement Approach for Health Supply Chain Management in Rwanda**

**Key Functional Areas**

- Resupply (quantification and forecasting)
- Ordering (procurement)
- Warehousing and inventory management
- Distribution/transportation (Rwanda Medical Supply branches only)

**Cross-Cutting Areas**

- Electronic logistics management information system (e-LMIS)
  - e-LMIS utilization
  - Data analysis
  - Data utilization for decision-making
- Financing of supply chain activities at Rwanda Medical Supply branches and service delivery points
- Human resources
- Governance
- Communications and coordination (synergy between different services/departments and stakeholders)

The QMIA has 2 components: periodic supervisory visits and discussion sessions. The central level, consisting of the MOH and central medical stores, performs these 2 components. Then, the RMS branches conduct supervisory visits and discussion sessions at SDPs with support from GHSC-PSM. Between 2017 and 2021, the intervention included 30 RMS branches and 596 SDPs.

#### Conducting QMIA Supervisory Visits

During their supervisory visits, government and GHSC-PSM staff (1) measure the supply chain performance using defined key performance indicators (KPIs) (Table 1); (2) identify key performance gaps; (3) provide capacity-building in supply chain management of health commodities to health facilities; (4) monitor progress toward actions taken during the previous sessions to address gaps; and (5) ensure the correct use of management tools, including the e-LMIS. Supervisory visits are planned in advance, with precise logistical and technical instructions to be applied before the visit, on arrival at the site, and after the visit.

**TABLE 1.** Key Performance Indicators Measured During Quality Management Improvement Approach Supervisory Visits in Rwanda^a^

**Indicator Name**	**Indicator Measurement**	**Performance Target Measured by the Indicator**
Product availability	Is this product available at this facility on the day of visit? (Yes=1, No=0)	Percentage of availability of health commodities at the facility on the day of visit.
Inventory accuracy	e-LMIS current stock on hand data compared to physical count. (Matching=1, Not matching=0)	Percentage of products with stock accuracy on the day of visit.
Order line fill rate	Compare the quantity requested vs. quantity received in e-LMIS for previous month. (Matching=1, Not matching=0)	Percentage of items ordered that are received to determine whether an order is filled in the correct quantities with the correct products.
Expiration rate	Has this product expired at this facility within the last 3 months? (Yes=1, No=0)	Rate of expiration at the facility in the last 3 months.
Stock according to plan	Is this product stocked according to plan (minimum and maximum) on the day of visit?	Product with stock levels above the established minimum level and below the established maximum level.
Stock card updated	Product with stock card updated with all required information at the day of visit? (Yes=1, No=0)	All products managed should have an updated stock card.
Data triangulation	Compare the e-LMIS consumption with cases or beneficiaries in HMIS (quantities have to be entered). This is for malaria and family planning commodities only within the last month.	Consumption data of ACTs recorded in e-LMIS should match with number of malaria cases recorded in HMIS. For family planning, all consumptions recorded in e-LMIS should match with the beneficiaries in HMIS.
Accuracy of e-LMIS consumption data	Compare the e-LMIS consumption with consumption recorded into registers during the last month.	All products dispensed to patients must be recorded (this means all products dispensed to patients in each dispensing window are captured in e-LMIS).
On-time delivery	Was the order delivered on agreed date? Check if the promised delivery date and the shipped date matched. Applies to the last month.	Percentage of all orders delivered within the agreed delivery date.
Order completeness	Number of orders shipped versus number of orders received during last month.	Percentage of shipped orders received electronically.
Invoice generated	Invoice generated and received during last month.	Informs if the orders are fully processed in the system and the unit prices are updated (for malaria and essential medicines).

Abbreviations: ACT, artemisinin-based combination therapy; e-LMIS, electronic logistics management information system; HMIS, health management information system.

^a^These indicators are then aggregated at district and national levels.

#### Conducting QMIA Discussion Sessions

After the visits, discussion sessions are held semiannually within the district by RMS branches with SDPs at identified locations. Discussion sessions aim to solve issues identified during supervisory visits and provide collaborative learning opportunities by sharing experiences, building skills in areas that need improvement, and setting targets. The sessions aim to: (1) monitor improvement recommendations for supply chain performance, (2) review the current performance, (3) train and conduct problem solving, and (4) provide recommendations for continuous improvement. For each selected supply chain functional area, the process follows several steps (Table 2).

**TABLE 2.** Objectives, Inputs, and Outputs of Steps in the Quality Management Improvement Approach Discussion Sessions in Rwanda

**Steps**	**Objectives**	**Inputs**	**Outputs**
1. Define the problem	Understand and define the problem to be solved. Establish a goal for what needs to be achieved.	Something wrong or that could be improved.	A clear definition of the problem and opportunity with goals to fix it.
2. Brainstorm ideas	Brainstorm some of the ways to solve the problem, aiming to create a list of possible solutions to choose from.	A goal, research of the problem and possible solutions, imagination.	Picklist of possible solutions that would achieve the stated goal.
3. Decide on a solution	Find a solution that is effective (it will meet the goal), efficient (is affordable), and has the fewest side effects (limited consequences from implementation).	Picklist of possible solutions and decision-making criteria.	The decision of what solution will be implemented.
4. Implement the solution	Plan and execute the implementation, often iteratively (the focus should be on short implementation cycles with testing and feedback, not trying to get it “perfect” the first time).	Decision, planning, hard work.	Resolution of the problems.
5. Review the results	Review what worked, what did not, and what impact the solution had. It also helps to improve long-term problem-solving skills.	Resolutions, results of the implementation.	Insights, case studies, journal articles.

## RESULTS

The QMIA has proven to be a successful training and capacity-building platform for supply chain management in Rwanda. The team for this activity is composed of 10 trainers from the central level (Rwanda’s MOH and GHSC-PSM) who have trained 30 RMS branch managers, who in turn conduct training at SDPs and collect data during supervisory visits (Table 3). From 2017 to 2021, 1,296 members of the health supply chain staff participated in the QMIA.

**TABLE 3.** Participants in the Quality Management Improvement Approach in Rwanda

**Role in the QMIA**	**Role in the Supply Chain**	**Participants, No.**
Trainers	Central level supervisor	10
	RMS branch manager	30
Facilitators	Data quality field officer	30
Trainees	Hospital pharmacist	50
	SDP pharmacy store manager	596
	SDP data manager	580

Abbreviations: RMS, Rwanda Medical Supply; SDP, service delivery point.

QMIA has helped in many aspects of supply chain management, such as inventory accuracy, expiration rate, and stock according to plan, although challenges remain (e.g., data triangulation). From 2017 to 2019, the program contributed to significant improvements in several key supply chain outcomes: recording of commodity consumption improved from 35% to 95%, e-LMIS utilization improved from 55% to 96% (although some barriers remain at SDPs primarily due to lack of equipment or skills to operate the e-LMIS), the accuracy of inventory data improved from 25% to 85%, and stock-outs for all types of commodities were reduced from 10% to 1%.

QMIA has helped in many aspects of supply chain management, such as inventory accuracy, expiration rate, and stock according to plan, although challenges remain.

**FIGURE 3:**
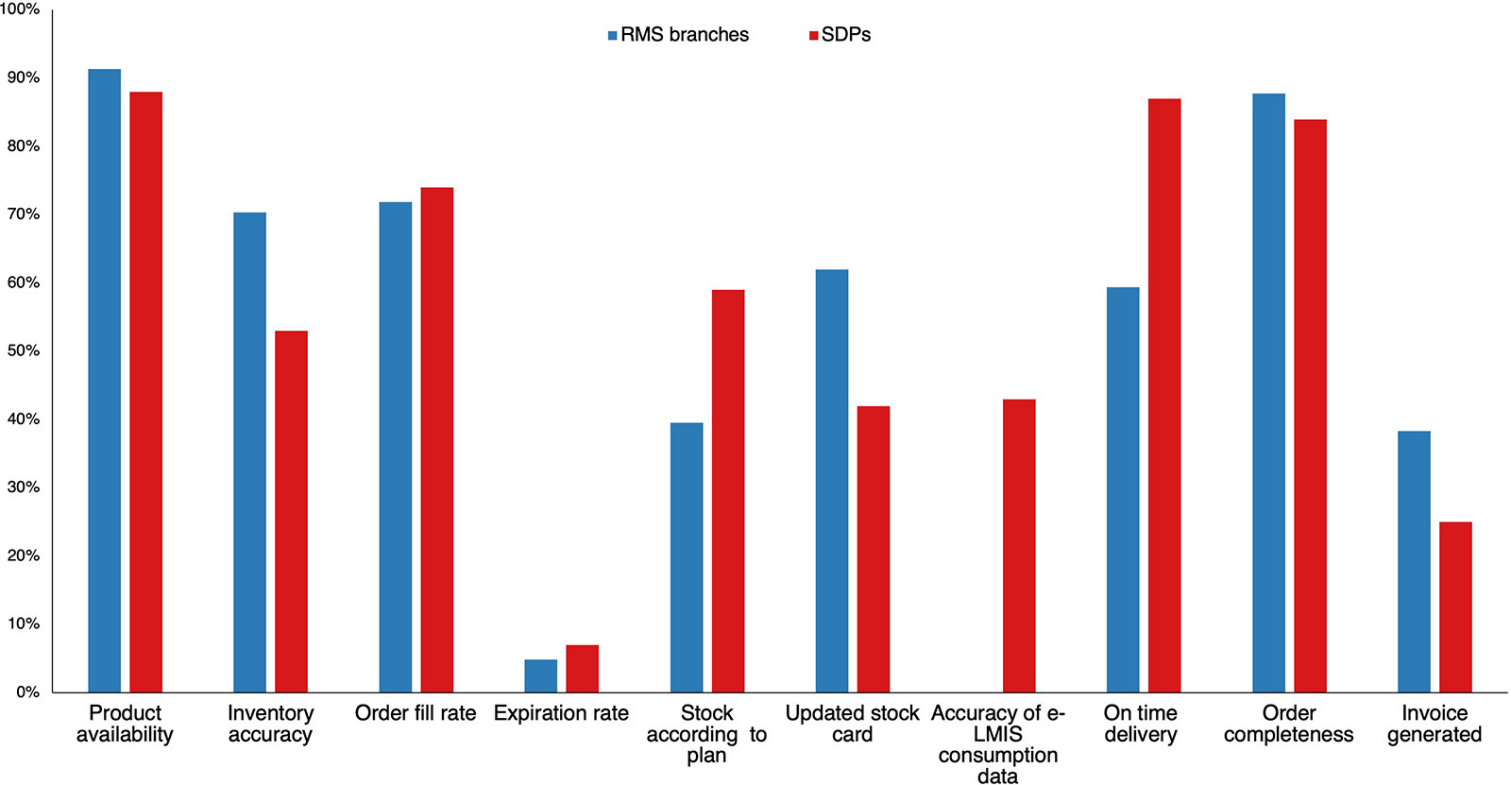
Overall Quality Management Improvement Approach Key Performance Indicators at RMS Branches and SDPs^a,b^ Abbreviations: e-LMIS, electronic management information system; RMS, Rwanda Medical Supply; SDP, service delivery point. ^a^Data from May 2021. ^b^The indicator “Accuracy of e-LMIS consumption data” was only measured in SDPs, since RMS branches do not directly treat patients.

**FIGURE 4:**
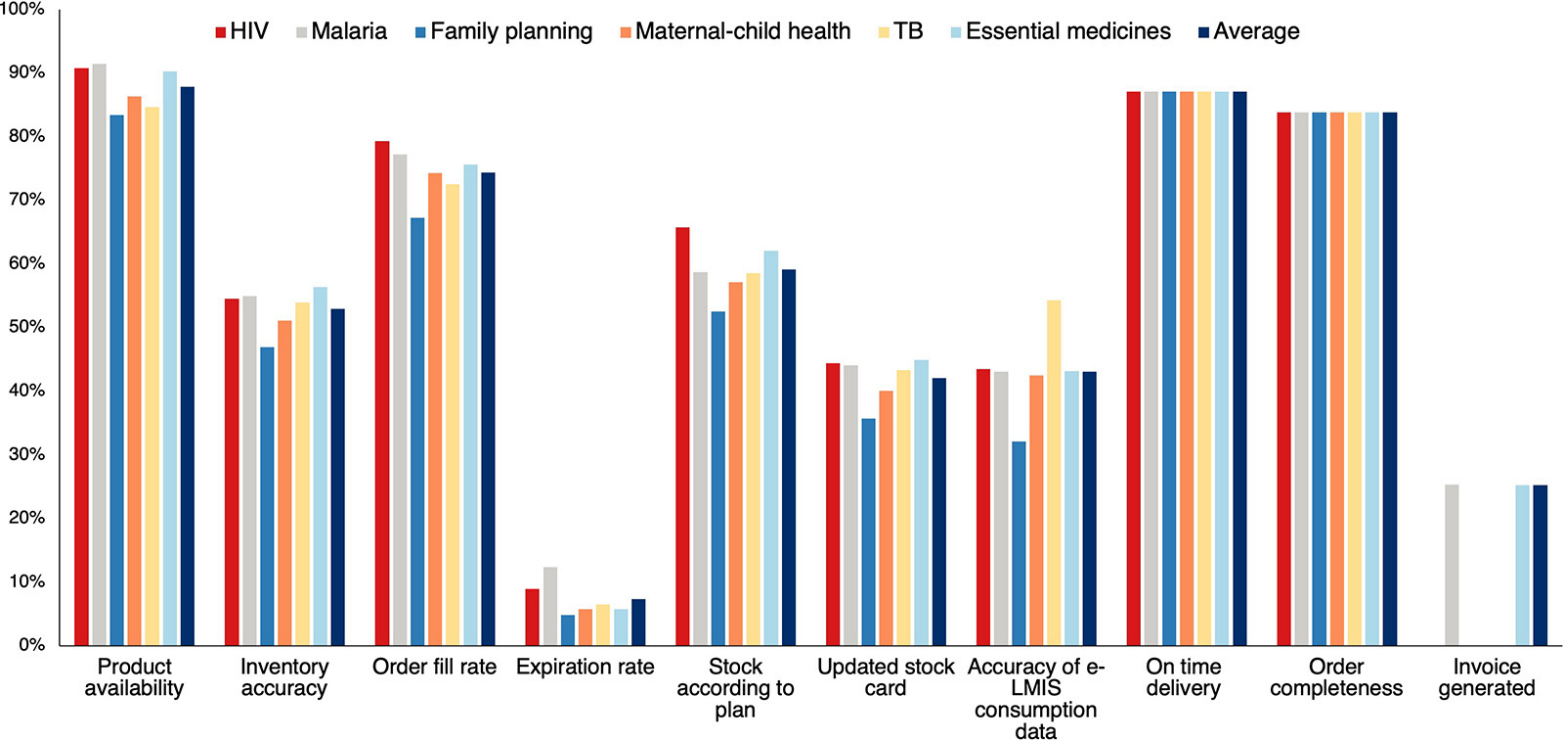
Overall Quality Management Improvement Approach Key Performance Indicators By Program Using Data From RMS Branches^a,b^ Abbreviations: e-LMIS, electronic management information system; RMS, Rwanda Medical Supply. ^a^Data from May 2021. ^b^The indicator “Invoice generated” was only applicable to some of the programs; for others there was no invoicing when the site visit was conducted.

QMIA KPIs at different RMS branches and SDPs are shown in Figure 3. Product availability was the KPI with the best performance both at RMS branches and SDPs, followed by order completeness at RMS branches and on-time delivery at SDPs (all >80%). Expiration rate and invoice generated were the indicators with the lowest performance (<40%), while data triangulation could not be calculated in many cases because of discrepancies between medicines dispensed to patients versus patient numbers and/or improper recordkeeping. KPIs by program followed a similar pattern (Figure 4). We did not find trends for specific districts whose SDPs were performing particularly good or bad (data not shown).

Notably, the QMIA effectively addressed performance gaps created by the supply chain staff turnover and staff rotation within an SDP. Thus, the upgrades contributed to greater system efficiency. Although these results are expected to lead to higher patients’ satisfaction, specific data for this outcome have not been collected yet, and there are plans to include this outcome among KPIs in the future.

The program also improved communication between supply chain workers at different levels. For example, facilitators created a WhatsApp group including all district personnel and another between each central team with the district representative. Using social media platforms such as WhatsApp to communicate about performance and resolve issues has fostered a positive behavioral change. This is contributing to the development of local solutions to address challenges in the supply chain operation and management.

However, in 2020, QMIA was not implemented because of disruptions caused by the COVID-19 pandemic, including lockdowns, travel restrictions across districts, and supply chain staff’s efforts shifting to COVID-19 prevention and control activities. Rwanda’s drug supply system was interrupted by the emergence of the pandemic, primarily because of the limited importation of goods from abroad (the country’s pharmaceutical sector heavily relies on imports, mainly from China and India) and the panic-buying practice among customers and some institutions.^15^ The prices of different drugs increased and some local pharmaceutical companies shifted their manufacturing capacity to infection prevention–related products such as hand sanitizers or face masks. All these factors led to a shortage of some products.^15^ As a result of the pandemic-related disruptions to the QMIA program, there was a reduction in KPIs: e-LMIS utilization decreased to 88% and inventory accuracy to 70% in 2021. With the subsequent reduction of COVID-19 cases and an increased number of people vaccinated, supply chain staff have since resumed their efforts to ensure services are provided at RMS branches and SDPs. This has since shifted both staffing numbers and KPIs to the previous numbers as travel and restrictions were lifted.

During supervisory visits conducted until 2021, the main challenges detected in RMS branches included shortage of inventory for some essential medicines; confusion over the new scope of RMS branches to support health facilities; lack of standard operating procedures for data triangulation, particularly for malaria and family planning commodities; and stock-out of essential medicines. Most of the challenges detected in 2021 were largely manageable and required refresher trainings to staff, which were already provided. Regarding the lack of inventory for some essential medicines, the main cause was delayed use of order forms, leading to stock-outs. This has been solved by training staff on timely use of order forms.

The main challenges identified at health facilities during supervisory visits included high turnover of store managers in charge of the health facility pharmacy (to solve this, the MOH is currently designing incentives that will foster staff retention and motivation, such as trainings, promotions to high performers, and better remunerations); lack of dedicated staff to manage pharmacy operations; and insufficient time allocated to pharmacy-related activities. Additional challenges included a lack of the following: training in inventory management and e-LMIS use for new staff; harmonized dispensing register and information and technology infrastructure needed to perform pharmacy-related activities; an automated process for recording consumption; standard operating procedures for data triangulation, particularly for malaria and family planning commodities; and key information on family planning registers from community health workers. These challenges have been addressed by providing refresher training to staff so they can become more conversant with using the e-LMIS or other technology infrastructure and by upgrading the technology infrastructure to be more user friendly and better meet users’ demands. Finally, the time-consuming procurement of commodities outside of RMS branches is being streamlined so that SDPs can easily access the necessary medicines at every RMS branch.

## LESSONS LEARNED

The main lesson learned from the application of the QMIA was that continuous capacity-building is critical to a well-performing supply chain. In addition, the recommendations from training and supervisory visits have provided a better understanding of the reasons behind the poor performance. One of the lessons learned from the impact of the COVID-19 pandemic on supply chain operations was the need to have electronic tools for continuous capacity-building, such as online learning platforms. As a solution to disruptions caused by the pandemic, the MOH and GHSC-PSM jointly developed virtual supply chain training modules and some of the QMIA in-person sessions were conducted online. These materials continue to be used to train supply chain staff at all levels and strengthen the workforce’s capacity at SDPs and regional and central medical stores. In this way, the staff learns about supply chain best practices at their workstations, saving time and resources from supervision and attendance at workshops. However, in-person QMIA sessions will also be conducted in the future to continue capacity-building and improve data quality along the supply chain.

The main lesson learned from the application of the QMIA was that continuous capacity-building is critical to a well-performing supply chain.

**FIGURE 5:**
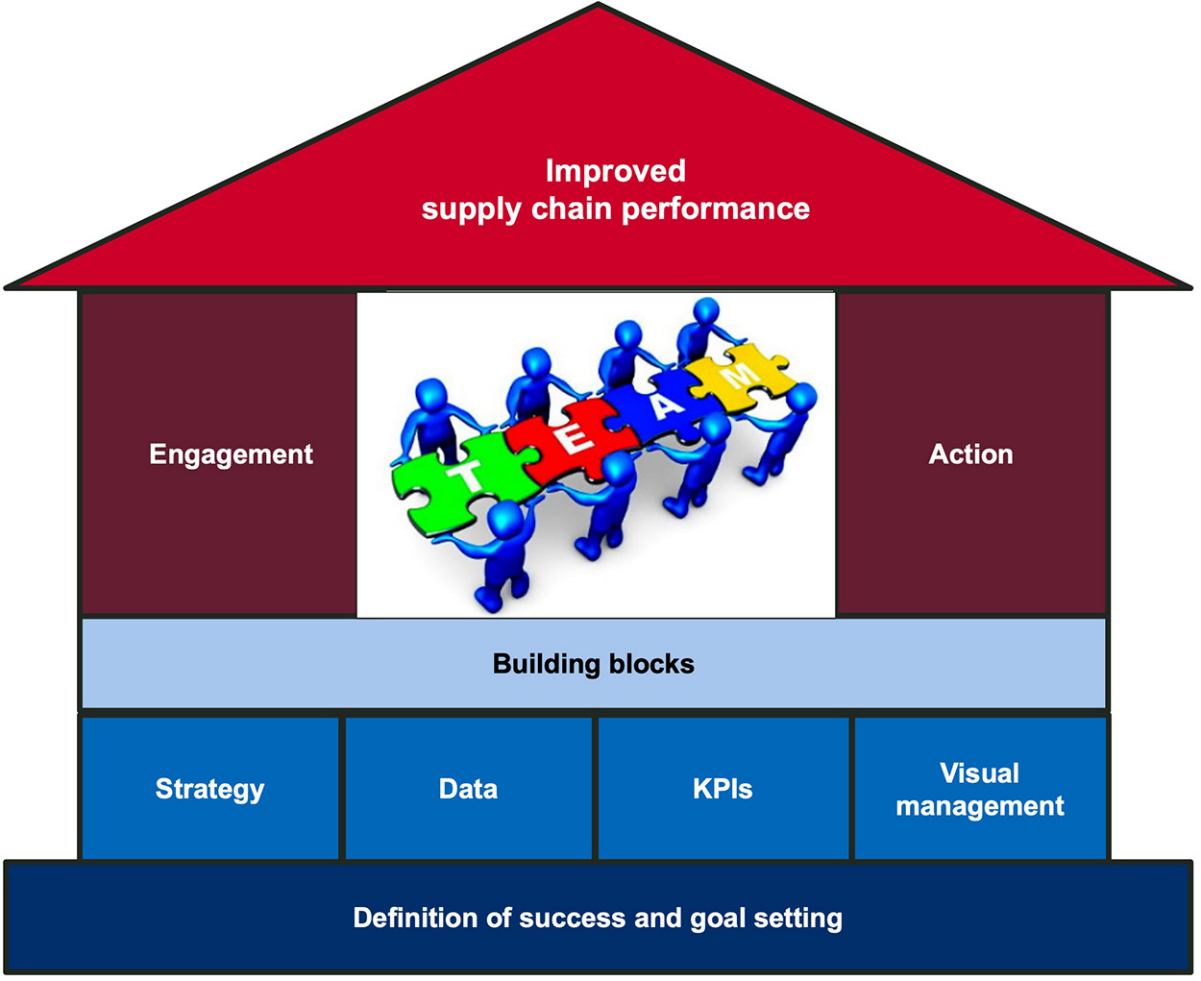
Building Blocks, Successes, and Lessons Learned: How the Quality Management Improvement Approach Contributed to Supply Chain Improvement in Rwanda^a^ Abbreviation: KPI, key performance indicator. ^a^ First, the goals and success of the activity were clearly defined. Then, the strategy, data, KPIs, and visual data management were used as building blocks to promote team engagement and action to solve challenges in supply chain operation. This resulted in improved supply chain performance.

We also learned there was a critical need at the SDPs for staff with supply chain backgrounds and experience and that supply chain professionals need a career development plan that promotes motivation and professional growth. In this sense, the selection and provision of adequate training is crucial. Non-accredited training (i.e., training not recognized by the MOH, the World Health Organization, or other accredited bodies) should be excluded since it cannot contribute to professional growth in the supply chain workforce. The main building blocks, successes, and lessons learned are summarized in Figure 5.

### Strengths and Limitations

The main strength of the QMIA implementation lies in its establishment of a process of continuous improvement and knowledge transfer, where the central level trains RMS branches and RMS branches train SDPs. Another important point is the government ownership of the QMIA, which is reinforced through collaboration with development partners. These interventions are fully institutionalized, being adopted by the MOH and RMS branches under the technical assistance of GHSC-PSM. After the project ends in 2023, the MOH will assume responsibility for QMIA implementation by providing financial support to be applied independently by RMS. The MOH will continue to carefully evaluate the impacts and challenges that QMIA faces through supervisory visits and discussions and may decide to focus on the most problematic RMS branches or SDPs.

The main strength of the QMIA implementation lies in its establishment of a process of continuous improvement and knowledge transfer, where the central level trains RMS branches and RMS branches train SDPs.

The main limitations of this approach have been observed at the SDP level because a majority of supply chain staff do not have an academic background in supply chain management (most of them are nurses), and there is a limited number of supply chain cadres. To address this challenge, the MOH, through the Human Resource for Health Secretariat, is ensuring the availability of enough trained staff to manage the health supply chain.

Another challenge is the increased workload due to limited staff handling multiple tasks at SDPs. This is being addressed by allocating enough funds to district hospitals to hire staff to be sent to the SDPs, hence reducing the workload of existing staff.

Other supply chain–related interventions conducted in the last few years in Rwanda, including quantification, stock status monitoring, and other capacity-building sessions for RMS and MOH staff, may have also influenced the observed results.

## RECOMMENDATIONS TO BUILD RESILIENCE

Given the noticeable improvement in crucial supply chain outcomes with QMIA implementation, we recommend that other countries facing similar challenges in supply chain management adopt this approach to improve the performance of supply chain processes at different levels.

Between 2017 and 2021, QMIA supervision detected performance gaps in supply chain management at both RMS branches and SDPs and proposed the following recommendations that can help build resilience at different levels.

### MOH

- Support health facilities to have a fully dedicated store manager with the knowledge to support supply chain operations.
- Improve interoperability of hospital systems (e-LMIS and hospital management systems, such as electronic medical records) to ensure unique entry of data and minimize efforts.
- Reinforce data validation through data triangulation between RMS branches and district hospitals to ensure that planning is based on accurate information.

### RMS Branches

- Increase the availability of essential medicines at RMS headquarters and branches.
- Build capacity of health facility staff on stock management of health commodities and tools (e.g., e-LMIS, stock cards, etc.).
- Develop a capacity-building plan for new staff at RMS branches and SDPs.
- Train several staff members to manage pharmacy operations to address gaps created by staff turnover.
- Monitor the performance of supply chain operations at SDP level.
- Reinforce data validation through data triangulation between RMS branches and district hospitals.

### SDPs

- Maintain motivated full-time staff dedicated to supply chain management at the SDP level.
- Record consumptions on a regular basis in the e-LMIS.
- Conduct a monthly data quality meeting at SDPs before reporting (including data and store managers, head of facility, and monitoring and evaluation staff).
- Ensure that product data are correctly recorded and promptly reported, in collaboration with data managers, the service responsible, and community health workers.
- Reinforce data validation between the data manager, laboratory technicians, and store managers at health facilities.

These recommendations are in the context of QMIA activity in Rwanda, but other countries willing to apply this approach need to first identify the main gaps and evaluate the performance of the supply chain management and human resources in their own context. Some of the enablers for the success of the QMIA in Rwanda include having policies and strategies that reinforced the national commitment to health and development (e.g., policies promoting the digitalization of the health sector) and the existence of institutions that support the health sector, such as RMS and the Rwanda Biomedical Center.

## CONCLUSIONS

The Rwanda MOH and GHSC-PSM implemented the QMIA workforce development intervention to overcome challenges identified in the supply chain of health commodities. Through the QMIA, supply chain professionals in Rwanda have received successful training and capacity-building, which has contributed to significantly improved key supply chain outcomes. This approach can be implemented in other settings having issues with end-to-end data visibility and with supply chain personnel operation and communications.

## Notes

**Peer Reviewed**

**First published online:** January 18, 2023.
